# Procyanidin B2 Activates PPARγ to Induce M2 Polarization in Mouse Macrophages

**DOI:** 10.3389/fimmu.2019.01895

**Published:** 2019-08-07

**Authors:** Ying Tian, Chunmiao Yang, Qinyu Yao, Lei Qian, Jia Liu, Xinya Xie, Wen Ma, Xin Nie, Baochang Lai, Lei Xiao, Nanping Wang

**Affiliations:** ^1^Cardiovascular Research Center, School of Basic Medical Sciences, Xi'an Jiaotong University, Xi'an, China; ^2^The Advanced Institute for Medical Sciences, Dalian Medical University, Dalian, China; ^3^College of Basic Medical Sciences, Dalian Medical University, Dalian, China

**Keywords:** procyanidin B2, macrophages, macrophage polarization, peroxisome proliferator-activated receptor γ, gene regulation

## Abstract

Procyanidins, a subclass of flavonoids found in commonly consumed foods, possess potential anti-inflammatory activity. Manipulation of M1/M2 macrophage homeostasis is an effective strategy for the treatment of metabolic inflammatory diseases. The objective of this study was to determine the effect of procyanidins on macrophage polarization. Procyanidin B2 (PCB2), the most widely distributed natural procyanidins, enhanced the expressions of M2 macrophage markers (Arg1, Ym1, and Fizz1). PCB2 activated peroxisome proliferator-activated receptor γ (PPARγ) activity and increased the expressions of PPARγ target genes (CD36 and ABCG1) in macrophages. Inhibition of PPARγ using siRNA or antagonist GW9662 attenuated the PCB2-induced expressions of M2 macrophage markers. In addition, we identified cognate PPAR-responsive elements (PPREs) within the 5'-flanking regions of the mouse Arg1, Ym1, and Fizz1 genes. Furthermore, macrophages isolated from db/db diabetic mice showed lower expressions of M2 markers. PCB2 effectively restored the Arg1, Ym1, and Fizz1 expressions in a PPARγ-dependent manner. These findings support the notion that PCB2 regulated macrophage M2 polarization *via* the activation of PPARγ. Our results provide a new mechanism by which procyanidins exert their beneficial anti-inflammatory effects.

## Introduction

Macrophages are key cellular components of innate immunity, acting as a main player in the first-line defense against pathogens and the modulation of immunological homeostasis (1). In response to various environmental signals (e.g., activated lymphocytes, damaged cells, and microbial products) or different pathophysiologic stimuli, macrophages acquire distinct functional phenotypes *via* undergoing different phenotypic polarization (classical M1 activation or alternative M2 activation) (2). Stimulated by lipopolysaccharides (LPS), interferon-γ or tumor necrosis factor-α (TNF-α), M1 macrophages are characterized by high antigen presentation and expressions of pro-inflammatory cytokines [e.g., interleukin (IL)-1β, IL-6, and the cell membrane molecule CD86], playing an important role in host defense against infection (3). In contrast, M2 macrophages are characterized by expression of distinct marker genes such as arginase-1 (Arg1), found in inflammatory zone 1 (Fizz1), chitinase-3-like protein 3 (Ym1), and mannose receptor (CD206), exerting anti-inflammatory and tissue repairing effects (4).

Macrophage M1/M2 polarization is a tightly controlled process involving a set of molecular signaling pathways as well as transcriptional and/or post-transcriptional regulatory networks (5). Nuclear factor-kappa B (NF-κB), signal transducer and activator of transcription 1 (STAT1), CCAAT/enhancer binding proteins α (C/EBP-α), C/EBP-δ, interferon regulatory factor 9 (IRF9), and Krüppel-like factor 6 (KLF6) are important transcription factors involved in M1 polarization (6–9), whereas STAT3, STAT6, C/EBP-β, IRF4, KLF4, GATA binding protein 3 (GATA3), and peroxisome proliferator-activated receptors (PPARs) are associated with M2 polarization (10–14). Disturbed macrophage polarization is implicated in the development of metabolic inflammatory diseases such as obesity, type 2 diabetes mellitus (T2DM) and cardiovascular diseases (15). An imbalance in the ratio of M1/M2 macrophages, with enhanced “pro-inflammatory” M1 macrophages or/and impaired “anti-inflammatory” M2 macrophages is a feature of metabolic inflammation (16). Therefore, reshaping macrophage polarization could be a potential strategy against metabolic inflammatory diseases.

Procyanidins are members of the flavonoids found in many plant foods such as apples, cocoa beans, grape seed, and red wines (17). The most common procyanidins are the B-type procyanidins (PCB) (18). Epidemiological evidence suggested that consumption of procyanidins reduced the risk of cardiovascular diseases, T2DM and cancers (19). The anti-inflammatory and anti-oxidative properties might contribute to the health benefits of procyanidins (20). However, the molecular mechanisms underlying their anti-inflammatory effects remain incompletely understood. In the present study, we sought to examine whether PCB2 has an effect on macrophage polarization.

## Materials and Methods

### Reagents

Procyanidin B2 (PCB2) was purchased from MedChem Express (Monmouth Junction, NJ, USA). Rosiglitazone (RGZ) and GW9662 were purchased from Sigma-Aldrich (St. Louis, MO, USA). GW6471 and GSK0660 were purchased from Cayman Chemical (Ann Arbor, Michigan, USA). Anti-CD206 and anti-CD86 PE and PE-conjugated rat IgG2a, κ were from Biolegend (San Diego, CA, USA). Rabbit polyclonal antibodies against Ym1 and Fizz1 were purchased from Abcam (Cambridge, MA, USA). Phosphorylated PPARγ (Ser112) rabbit polyclonal antibody was purchased from Invitrogen (Carlsbad, CA, USA). PPARγ antibody was from Cell Signaling Technology (Danvers, MA, USA). Antibodies against β-actin, Arg1, and horseradish peroxidase (HRP)-conjugated secondary antibodies were from Santa Cruz Biotechnology (Santa Cruz, CA, USA).

### Isolation of Mouse Peritoneal Cavity Macrophages (PCMs)

We isolated PCMs from 12-week-old male C57BL/6J mice, diabetic db/db mice on a C57BL/KsJ background and non-diabetic littermate db/m^+^ mice as previously described (21). Briefly, 1 ml of 4% Brewer thioglycollate medium was injected into the peritoneal cavity. Three days later, the mice were sacrificed and peritoneal cavity was washed with ice-cold RPMI 1640 medium. The macrophages were collected from peritoneal fluid washes by refrigerated centrifuge at 400 × g for 10 min. The cells were resuspended and plated for 6 h. Attached cells were rinsed and cultured for 48 h before further treatment.

### Cell Culture and Treatment

Mouse monocytic cell line RAW264.7 and human embryonic kidney epithelial cell line HEK293 were from American Type Culture Collection (ATCC) and cultured in Dulbecco's modified Eagle medium (DMEM, Gibco) containing 10% fetal bovine serum (FBS, Gibco) with penicillin (100 U/ml) and streptomycin (100 U/ml) in 37°C with 5% CO_2_. Cells were treated with indicated concentrations of PCB2 (0.1–10 μM) for 24 h in serum-free DMEM medium. For all experiments, control cells treated with dimethylsulfoxide (DMSO, vehicle of the PCB2) were included.

### Quantitative Reverse Transcriptase-PCR (qRT-PCR)

Total RNA was isolated using TRIzol (Invitrogen), converted to cDNA by using iScript cDNA synthesis kit (Bio-rad). Real-time PCR was performed by using SYBR-green dye and *Taq* polymerase with an ABI 7500 real-time PCR System (applied biosystems). Quantification was calculated using the comparative threshold cycle (Ct) method and efficiency of the RT reaction (relative quantity, 2^−ΔΔCt^). Primers used in qRT-PCR were shown in Supplementary Table 1.

### Protein Extraction and Western Blot Analysis

Total proteins were extracted with lysis buffer (50 mM Tris-HCl pH 7.4, 100 mM NaCl, 15 mM EGTA, 0.1% TritonX-100, and protease inhibitor cocktail). Nuclear proteins were isolated with high-salt buffer (20 mM Tris-HCl, 1.5 mM MgCl_2_, 420 mM NaCl, 10% glycerol, 0.2 mM EGTA). Protein concentrations were determined with the BCA protein assay kit (Thermo Scientific). Protein samples were separated on 12% SDS-PAGE and transferred onto polyvinylidene difluoride (PVDF) membranes. Blots were incubated with specific primary antibodies at 4°C overnight and HRP-conjugated secondary antibodies for 1 h, and visualized by using the Enhanced chemiluminescence (ECL) System. β-actin was used as a loading control. Quantification of scanned images was performed with Image J software from NIH Image.

### Plasmids Transfection and Reporter Assay

HEK293 cells were transfected with the plasmids expressing PPARγ, PPRE-TK-luciferase reporter containing three copies of PPAR-response elements (PPRE) from acyl CoA oxidase (ACO) gene and β-galactosidase (β-gal) by using Lipofectamine 2000 (Invitrogen). After the treatments with PCB2 (0.1–10 μM) or RGZ (10 μM) for 24 h, cell lysates were harvested to measure the luciferase and β-gal activities. Luciferase activities were normalized to β-gal activity.

### Adenoviral Vectors and Infection

For adenoviral infection, RAW264.7 cells were incubated with recombinant adenoviruses encoding PPARγ (Ad-PPARγ) and Ad-tTA (an adenovirus expressing tTA, a tetracycline-responsive transactivator) in the presence or absence of tetracycline (0.1 μg/ml, a tet-off expression) for 48 h as previously described (22).

### Flow Cytometry Analysis

Cells were harvested and washed with cold PBS, incubated with anti-CD86 or CD206 PE, and PE-conjugated rat IgG2a, κ served as an isotype control for 30 min on ice in the dark, then analyzed by flow cytometry (FACSCalibur, BD Biosciences). Data was analyzed by FlowJo software (Tree Star Inc., Ashland, OR, USA).

### Chromatin Immunoprecipitation (ChIP) Assay

Cells were cross-linked with 0.75% formaldehyde, harvested, and sonicated on ice. Sheared chromatin was immunoprecipitated with anti-PPARγ or control IgG and protein A/G Sepharose beads. After washing, the immunoprecipitates were eluted, digested with proteinase K and extracted for DNA. DNA samples were amplified with primers flanking the putative PPREs in the regulatory regions of the mouse Ym1, Arg1, Fizz1 genes (the sequences are shown in Supplementary Table 2). Relative DNA binding was expressed as fold enrichment above the control IgG-immunoprecipitated samples.

### RNA Interference

The siRNA sequence targeting mouse PPARγ was as follows: 5′-GGGCGAUCUUGACAGGAAATT-3′ (sense) and 5′-UUUCCUGUCAAGAUCGCCCTT-3′ (antisense). The siRNA with scrambled sequence was used as negative control (NC siRNA). The double-stranded RNAs (100 nM) were transfected into RAW264.7 cells with Lipofectamine RNAi MAX (Invitrogen).

### Statistical Analysis

Results are shown as the mean ± standard error of the mean (SEM). Student's *t* test was performed to determine statistical differences between two groups and one-way ANOVA for multiple comparisons using the SPSS 16.0 (SPSS software, IBM, USA). *P* < 0.05 was considered statistically significant.

## Results

### PCB2 Promoted Macrophage M2 Polarization and Suppressed M1 Polarization

**Figure 1:**
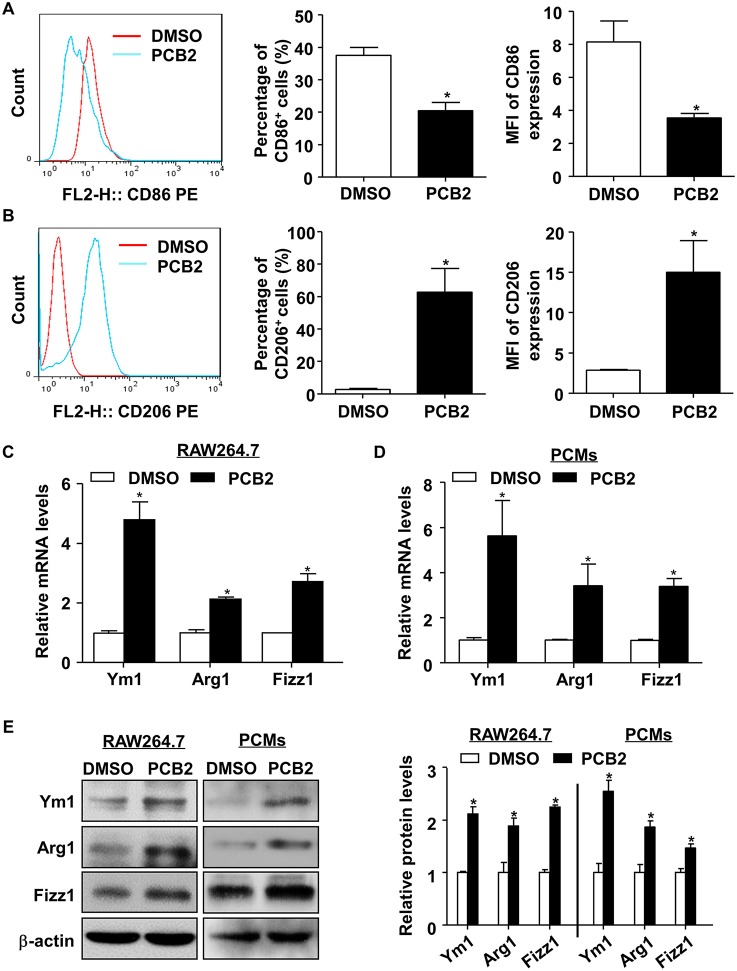
PCB2 promoted macrophage M2 polarization. RAW264.7 cells were incubated with PCB2 (10 μM) for 24 h and DMSO was used as vehicle control. The flow cytometry analysis was performed to evaluate the expressions of CD86 **(A)** and CD206 **(B)**. Representative histograms were obtained by flow cytometry analysis. Percentage of CD86^+^ or CD206^+^ macrophages and mean fluorescence intensity (MFI) of CD86 or CD206 expression on macrophages were quantified. RAW264.7 cells **(C)** and PCMs isolated from C57BL/6J mice **(D)** were incubated with PCB2 (10 μM) for 24 h. Total RNA was extracted and subjected to qRT-PCR for the assessment of Ym1, Arg1, and Fizz1 levels. **(E)** Cells lysates were analyzed for the protein levels of Ym1, Arg1, and Fizz1 by using western blotting in RAW264.7 cells and PCMs. Quantification of Ym1, Arg1, and Fizz1 protein levels. Data were shown as mean ± SEM, *n* = 3–6, ^*^P < 0.05 vs. vehicle (DMSO).

To study the effect of PCB2 on macrophage polarization, we first evaluated the cytotoxicity of PCB2 (Figure S1A) on RAW264.7 cells and PCMs from C57BL/6J mice by using the MTT assay. Cells were treated with PCB2 at various concentrations (0–10 μM) for 24 h. As shown in Figure S1B, PCB2 caused no decrease in cell viability in both RAW264.7 and PCMs. Therefore, we used PCB2 at a concentration of 10 μM to investigate the effects of PCB2 on macrophage polarization. We determined the percentage of CD86^+^ (a M1 marker) and CD206^+^ (a M2 marker) macrophages in the total cell population by using flow cytometry. As shown in Figures 1A,B, PCB2 diminished M1 phenotype and promoted polarization to an M2 phenotype in RAW264.7 cells. We then examined whether PCB2 increased the mRNA expression of M2 macrophage marker genes (Ym1, Arg1, and Fizz1). As shown in Figures 1C–E, PCB2 significantly increased the expressions of Ym1, Arg1, and Fizz1 both on mRNA and protein levels in RAW264.7 cells as well as in PCMs. Further, we treated RAW264.7 cells with PCB2 before the exposure to LPS and found that PCB2 inhibited LPS-stimulated expression of IL-6 and TNF-α, the M1 marker genes (Figure S2). Taken together, these results indicated that PCB2 promoted M2 macrophages polarization.

### PCB2 Activated PPARγ

**Figure 2:**
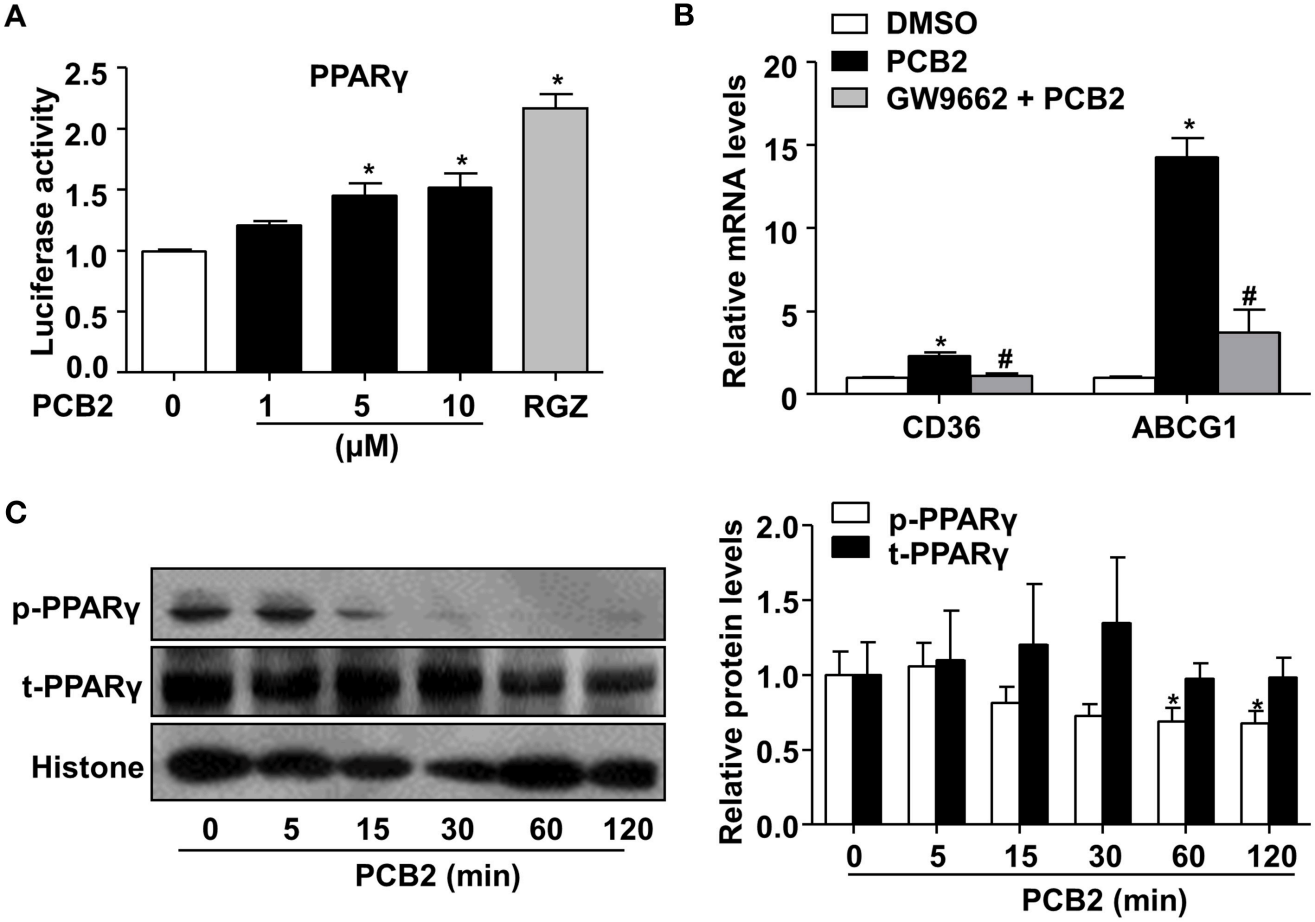
PCB2 activated PPARγ in RAW264.7 cells. **(A)** HEK293 cells were transfected with PPRE-luc and PPARγ plasmids and then treated with PCB2 (0–10 μM) or RGZ (10 μM, positive control) for 24 h. The luciferase activities were expressed as fold changes (*n* = 3). **(B)** RAW264.7 cells were pre-treated with GW9662 (5 μM) for 1 h, then exposed to PCB2 (10 μM) for 24 h, mRNA levels of CD36 and ABCG1 were measured by qRT-PCR (*n* = 4–6). **(C)** RAW264.7 cells were treated with PCB2 (10 μM) for 0–120 min, then nuclear extracts were analyzed for total and phosphorylated-PPARγ levels by using western blotting. Quantification of p-PPARγ and t-PPARγ levels in RAW264.7 cells (*n* = 6). Data were shown as mean ± SEM, ^*^P < 0.05 vs. vehicle; ^#^*P* < 0.05 vs. PCB2.

Activation of PPARγ potentiated the polarization of circulating monocytes to macrophages of the M2 phenotype (23). Thus, we examined whether PCB2 activated PPARγ activity. HEK293 cells were transfected with PPARγ expression and the PPRE-driven luciferase reporter plasmids before the exposure to PCB2. The reporter assay showed that PCB2 increased the PPARγ activity (Figure 2A). Further, we examined the effects of PCB2 on the expressions of the endogenous PPARγ target genes, such as cluster of differentiation 36 (CD36) and ATP binding cassette subfamily G member 1 (ABCG1) in RAW264.7 cells. As shown in Figure 2B, mRNA levels of CD36 and ABCG1 were increased by PCB2. However, GW9662, a selective antagonist of PPARγ, effectively abrogated the induction of CD36 and ABCG1 by PCB2, suggesting a PPARγ-specific mechanism.

Phosphorylation-mediated inhibition of transcriptional activity of nuclear receptors is an important “off-switch” of ligand-induced activity (24). It has been reported that phosphorylation of PPARγ by mitogen-activated protein kinase (MAPK) at serine 112 decreased ligand-binding affinity and affected coactivator recruitment (25). Thus, we investigated whether PCB2 inhibited the serine 112 phosphorylation of PPARγ to increase its activity in RAW264.7 cells. As shown in Figure 2C, PCB2 significantly decreased PPARγ phosphorylation with little effect on total protein level of PPARγ within the observed time periods. Taken together, we demonstrated that PCB2 inhibited PPARγ phosphorylation and activated PPARγ in macrophages.

### PCB2 Promoted Macrophage M2 Polarization via PPARγ Activation

**Figure 3:**
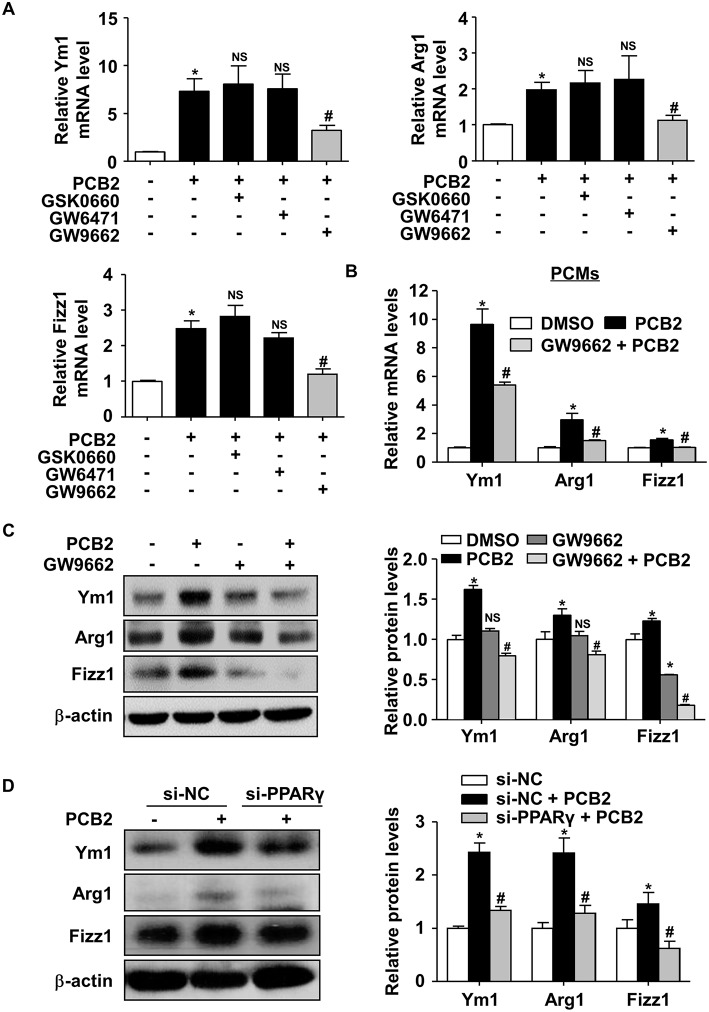
PCB2 promoted macrophage M2 polarization via PPARγ activation. **(A)** RAW264.7 cells were pre-treated with GSK0660 (2 μM), GW6471 (2 μM), or GW9662 (5 μM) for 1 h, then exposed to PCB2 (10 μM) for 24 h. Total RNA was extracted and subjected to qRT-PCR for the assessments of Ym1, Arg1, and Fizz1 mRNA levels. **(B)** PCMs were pre-treated with GW9662 (5 μM) for 1 h, then exposed to PCB2 (10 μM) for 24 h. Expression of Ym1, Arg1, and Fizz1 were measured by qRT-PCR. **(C)** Ym1, Arg1, and Fizz1 protein levels were measured by using western blotting. **(D)** RAW264.7 cells were transfected with PPARγ siRNA or NC siRNA for 24 h and then treated with PCB2 (10 μM) for another 24 h. Ym1, Arg1, and Fizz1 protein levels were detected by using western blotting. Data were shown as mean ± SEM, *n* = 3–5, ^*^P < 0.05 vs. control; ^#^*P* < 0.05 vs. PCB2 or NC siRNA treated with PCB2; NS, not significant.

To study whether PPARγ activity was required for the macrophage M2 polarization induced by PCB2, we pre-treated RAW264.7 cells with GW6471 (a selective PPARα antagonist), GSK0660 (a selective PPARδ antagonist), or GW9662 (a selective PPARγ antagonist) before the exposure to PCB2. As shown in Figure 3A, GW9662, but not GSK0660 or GW6471, significantly attenuated the effects of PCB2 on Ym1, Arg1, and Fizz1 mRNA levels. Experiments conducted in mouse PCMs also confirmed that PCB2 induction of Ym1, Arg1, and Fizz1 levels was attenuated by GW9662 (Figure 3B). GW9662 also abolished the PCB2 increased Ym1, Arg1, and Fizz1 at protein levels in RAW264.7 cells (Figure 3C). We also used the siRNA to silence the expression of endogenous PPARγ. As shown in Figure 3D, knockdown of PPARγ effectively diminished the induction of Ym1, Arg1, and Fizz1 by PCB2. Taken together, these results demonstrated that PCB2 promoted macrophages M2 polarization *via* a PPARγ-dependent manner.

### PPARγ Transcriptionally Activated Ym1, Arg1, and Fizz1 in RAW264.7 Cells

**Figure 4:**
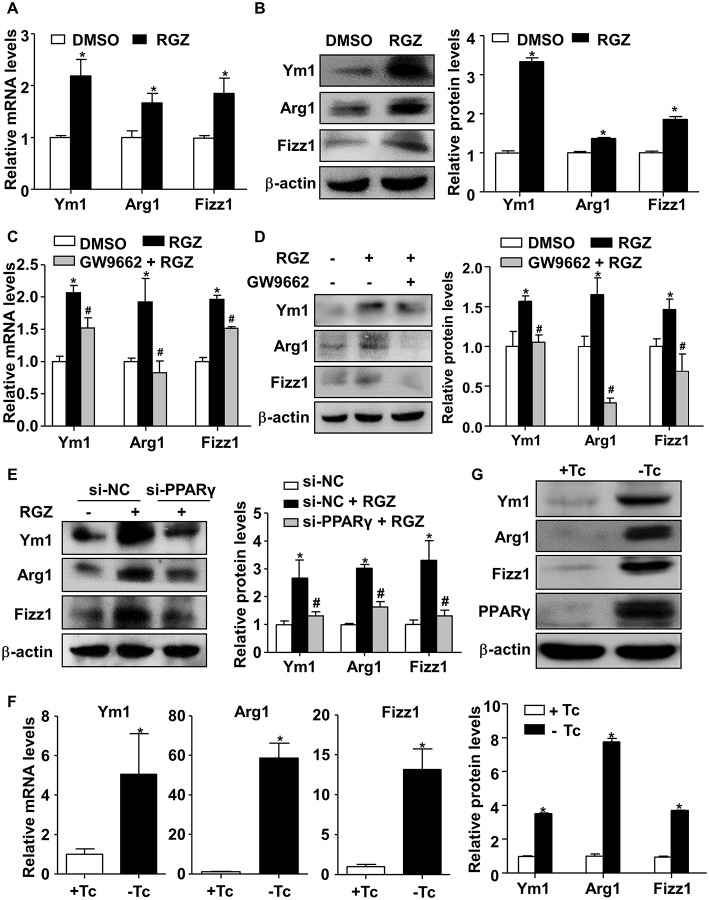
PPARγ transactivated Ym1, Arg1, and Fizz1 in RAW264.7 cells. **(A)** RAW264.7 cells were treated with RGZ (10 μM) for 24 h and DMSO as vehicle control. Total RNA was extracted and subjected to qRT-PCR for the assessment of Ym1, Arg1, and Fizz1 mRNA levels. **(B)** Ym1, Arg1, and Fizz1 protein levels were measured by using western blotting. **(C)** RAW264.7 cells were pre-treated with GW9662 (5 μM) for 1 h, then exposed to RGZ (10 μM) for 24 h. The mRNA levels of Ym1, Arg1, and Fizz1 were assessed by qRT-PCR. **(D)** The protein levels of Ym1, Arg1, and Fizz1 were measured by western blotting. **(E)** RAW264.7 cells were transfected with PPARγ siRNA or NC siRNA for 24 h, and then exposed to RGZ (10 μM) for 24 h. Protein levels of Ym1, Arg1, and Fizz1 were detected by using western blotting. **(F)** RAW264.7 cells were infected with Ad-PPARγ and Ad-tTA with or without Tc (0.1 μg/ml) for 48 h. The mRNA levels of Ym1, Arg1, and Fizz1 were assessed by qRT-PCR. **(G)** The protein levels of PPARγ, Ym1, Arg1, and Fizz1 were measured by western blotting. Data were shown as mean ± SEM, *n* = 3–4, ^*^P < 0.05 vs. control; ^#^*P* < 0.05 vs. RGZ or NC siRNA treated with RGZ.

To further examine how PCB2 induced the expressions of Ym1, Arg1, and Fizz1 *via* PPARγ, RAW264.7 cells were treated with RGZ, a specific agonist of PPARγ, for 24 h. As shown in Figures 4A,B, RGZ effectively induced Ym1, Arg1, and Fizz1 expressions at both mRNA and protein levels. To ascertain that RGZ acts *via* PPARγ, we treated RAW264.7 cells with GW9662 before the exposure to RGZ. As shown in Figures 4C,D, the RGZ increased Ym1, Arg1, and Fizz1 levels were significantly abrogated by GW9662. Knockdown of PPARγ also effectively diminished the induction of Ym1, Arg1, and Fizz1 by RGZ (Figure 4E). To further confirm the effects of PPARγ on Ym1, Arg1, and Fizz1 expressions, we adenovirally overexpressed PPARγ in RAW264.7 cells. As shown in Figures 4F,G, overexpression of PPARγ increased the expressions of Ym1, Arg1, and Fizz1 genes at both mRNA and protein levels.

### Identification of PPARγ-Binding Sites in the Ym1, Arg1, and Fizz1 Gene Promoters

**Figure 5:**
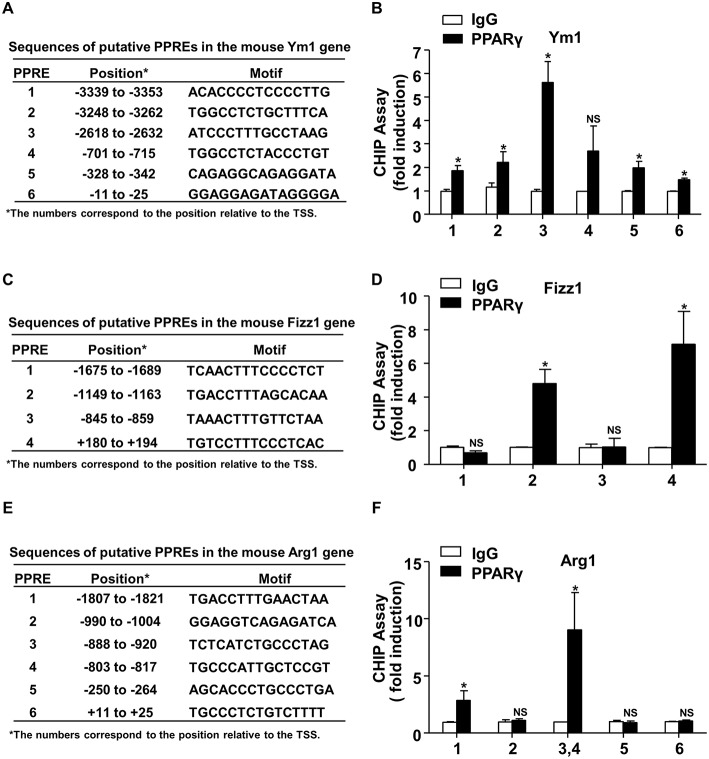
Identification of PPARγ-binding sites in the mouse Ym1, Arg1, and Fizz1 promoters. **(A,C,E)** Putative PPREs in the mouse Ym1, Fizz1, Arg1 gene promoters are listed with their positions in relation to TSS and core sequences. **(B,D,F)** ChIP assays were performed in RAW264.7 cells overexpressing PPARγ with the use of anti-PPARγ antibody or IgG as control. PPRE binding was quantified by using qPCR with the primers flanking the putative PPREs in the Ym1 **(B)**, Fizz1 **(D)**, Arg1 **(F)** gene promoters. The PCR results were expressed as fold change compared with IgG control. Data were shown as mean ± SEM, *n* = 3–8, ^*^P < 0.05 *vs*. IgG control; NS, not significant.

Next, we examined the mechanism by which PPARγ increased Ym1, Arg1, and Fizz1 expressions. Sequences analysis of the 5′-flanking regions of mouse Ym1, Fizz1, Arg1 genes by using our previously established prediction tool (http://www.ppargene.org) (26) and the online database of transcription factor binding profiles (http://jaspar.genereg.net) revealed multiple putative PPREs within the 4,000-bp regions upstream of the transcription start sites (TSS) (Figures 5A,C,E). ChIP assays showed that PPARγ could directly bind to the PPREs located at −3353/−3339 (PPRE1), −3262/−3248 (PPRE2), −2632/−2618 (PPRE3), −342/−328 (PPRE5), and −25/−11 (PPRE6) in the flanking region of the mouse Ym1 gene (Figure 5B), at −1163/−1149 (PPRE2), +180/+194 (PPRE4) in the mouse Fizz1 gene (Figure 5D), and at −1821/−1807 (PPRE1), −920/−888 (PPRE3), −817/−803 (PPRE4) in the mouse Arg1 gene (Figure 5F). These results indicated that Ym1, Arg1, and Fizz1 were direct targets of PPARγ in mouse macrophages.

### PPARγ Was Required for PCB2-Enhanced M2 Polarization in Diabetic Mice

**Figure 6:**
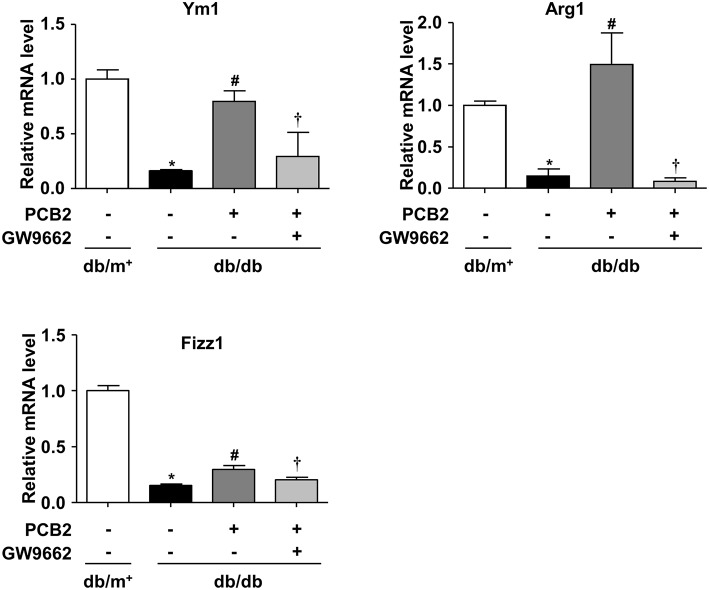
PCB2 ameliorated impaired M2 polarization via PPARγ activation. PCMs isolated from db/db or db/m^+^ mice were pre-treated with or without GW9662 (5 μM) for 1 h, then exposed to PCB2 (10 μM) for 24 h. The mRNA levels of Ym1, Arg1, and Fizz1 were measured by qRT-PCR. Data were shown as mean ± SEM, *n* = 6, ^*^P < 0.05 vs. db/m^+^ treated with vehicle; ^#^*P* < 0.05 vs. db/db treated with vehicle; ^†^*P* < 0.05 vs. db/db treated with PCB2.

Macrophages infiltration in vessel walls and adipose tissues is a pathological feature in atherosclerosis and obesity (27). To investigate whether PCB2 altered macrophage polarization in obese diabetic mice, we isolated PCMs from db/db mice and treated them with PCB2 for 24 h. As shown in Figure 6, compared with db/m^+^ control mice, M2 markers (Ym1, Arg1, and Fizz1) were down-regulated in PCMs from db/db mice. PCB2 significantly restored the downregulated M2 markers in db/db PCMs. However, the reversal of macrophage M2 polarization markers was abolished when the cells were pre-incubated with PPARγ antagonist GW9662.

## Discussion

In this study, we demonstrated that PCB2, a natural flavonoid, induced M2 macrophage polarization *via* the activation of PPARγ. This was supported by several lines of evidence: (1) PCB2 decreased the number of M1 macrophages and enhanced the expressions of M2 markers; (2) PCB2 activated PPARγ; (3) Both genetic and pharmacological inhibitions of PPARγ abrogated expressions of M2 markers induced by PCB2; (4) PPARγ transcriptionally activated Arg1, Ym1, and Fizz1 in macrophages; (5) PPARγ activation was required for PCB2 amelioration of the impaired M2 polarization in diabetic mice.

Procyanidins present in many plant foods, such as cinnamon, grape, cocoa beans, and apples (17). Fruit consumption has been associated with a reduced risk for cardiovascular disease both in Western and Chinese populations (28, 29). The protective effect was generally attributed, at least in part, to the anti-inflammatory activity of procyanidins in fruits. Our previous study showed that PCB2 inhibited the activation of NLRP3 inflammasome *via* the suppression of AP-1 pathway in endothelial cells (18). Terra et al. showed that procyanidins inhibited pro-inflammatory molecules C-reactive protein (CRP) and IL-6 expression whereas enhanced the expression of anti-inflammatory molecules, decreasing the low-grade metabolic inflammation *in vivo* (30). Byun et al. found that procyanidin C1 inhibited LPS-induced MAPK and NF-κB activations through toll-like receptor 4 (TLR4) in RAW264.7 cells and primary bone marrow-derived macrophages (BMDMs) (31). In the present study, we found that PCB2 suppressed pro-inflammatory macrophage M1 polarization and enhanced macrophage M2 polarization (Figure 1). The physiological concentrations of PCB2 are highly variable and depend on the dietary conditions and the techniques of detection (32–34). In human, plasma level of PCB2 reached 41 ± 4 nM at 2 h after the consumption of cocoa (35). Shoji et al. intragastrically administrated apple procyanidins to Wista rats and used HPLC-tandem MS to detect a plasma level of PCB2 as 17.6 ± 3.8 μM (36). In previous studies, PCB2 were used at 20–200 μM in U937, THP-1, RAW264.7, and HL-60 cell lines (37–40). Here, we used PCB2 at 10 μM, which is much higher than the plasma level in human after usual food intake but is achievable in animals.

Peroxisome proliferator-activated receptor γ (PPARγ) is one of a family of nuclear receptors that is responsible for regulating glucose homeostasis, cell differentiation, lipid metabolism, and inflammation (41). In addition, PPARγ activation is involved in M2 polarization (23). We attempted to determine whether PCB2 could exert anti-inflammatory through PPARγ activation. Here, we showed that PCB2 promoted M2 macrophage polarization mainly depending on PPARγ. This notion was supported by its capacity of activating the PPARγ-reporter and the induction of endogenous PPARγ target genes. More importantly, the effects of PCB2 on the induction of target genes and M2 markers were attenuated by PPARγ inhibition (Figure 2). PPARγ could be phosphorylated by MAPK, AMP-activated protein kinase (AMPK) or protein kinase C (PKC) (42, 43). In this study, we showed that PCB2 blocked PPARγ serine 112 phosphorylation, which might be one putative mechanism for the regulation of PPARγ activity by PCB2 (Figure 3). However, it remains to be examined whether this phytochemical serves as a *bona fide* ligand for PPARγ. PPARγ agonists are used to treat insulin resistance associated with metabolic syndrome and T2DM (44). By using AutoDock, a tool for virtual screening of molecular interactions, we found that PCB2 has a potential binding with PPARγ (data not shown).

In the present study, we also provided evidence that PPARγ activation is necessary for PCB2 induction of M2 polarization. PPARγ agonist and Ad-PPARγ increased the induction of Arg1, Ym1, and Fizz1 at both mRNA and protein levels. The induced expressions of Arg1, Ym1, and Fizz1 by PPARγ agonist were attenuated by inhibition of PPARγ (Figure 4). Furthermore, we identified Arg1, Ym1, and Fizz1 as direct targets of PPARγ (Figure 5). As a transcription factor, the primary mechanism for PPARγ to regulate gene expression is through its binding to specific PPRE in the regulatory regions of the target genes. In the present study, we found recurrent PPREs in the mouse Arg1, Ym1, and Fizz1 promoters and confirmed the PPARγ binding. Indeed, a previous study showed that Arg1 was regulated by PPARγ and involved in M2 polarization (23).

Chronic inflammation is an important pathological feature of obesity, T2DM as well as cardiovascular diseases (45). Resolving metabolic inflammation is one potential strategy to treat these metabolic disorders (46). Metformin (47) and thiazolidinediones (23) have been known to restrain low-grade inflammation. On the other hand, searching natural compounds modulating inflammation represents a promising approach for the treatment. Recently, several natural agents, such as lupeol (48), resveratrol (49), and geraniin (50), were reported to modulate macrophage polarization in several pathologic contexts. In the present study, expression of Arg1, Ym1, and Fizz1 was markedly decreased in PCMs isolated from diabetic db/db mice. Impaired expressions of M2 markers were significantly reversed by PCB2 treatment. However, GW9662 attenuated the effect of PCB2 in db/db PCMs (Figure 6). In various rodent models for diabetes, anti-diabetic effects of procyanidins have been reported, including their roles in insulin secretion and sensitivity, food intake, obesity, and inflammatory and oxidative responses (51). Previously, we reported that PCB2 attenuated NLRP3 inflammasome activation in endothelial cells (18). Since NLRP3 inflammasome activation and endothelial dysfunction are also important pathophysiological steps in diabetes and cardiovascular diseases, PCB2 may exert pleiotropic effects *in vivo*. Nevertheless, our *in vitro* and *ex vivo* findings demonstrated a macrophage-specific nutritional immunology action of procyanidins. However, it is worth noting that procyanidins can be converted into multiple metabolites through metabolism *in vivo* (52). Further study of the pharmacodynamics and kinetics of PCB2 may help understanding of the therapeutic actions and the pharmacological modes of PCB2 in the prevention and treatment of metabolic disorders.

## Conclusion

**Figure 7:**
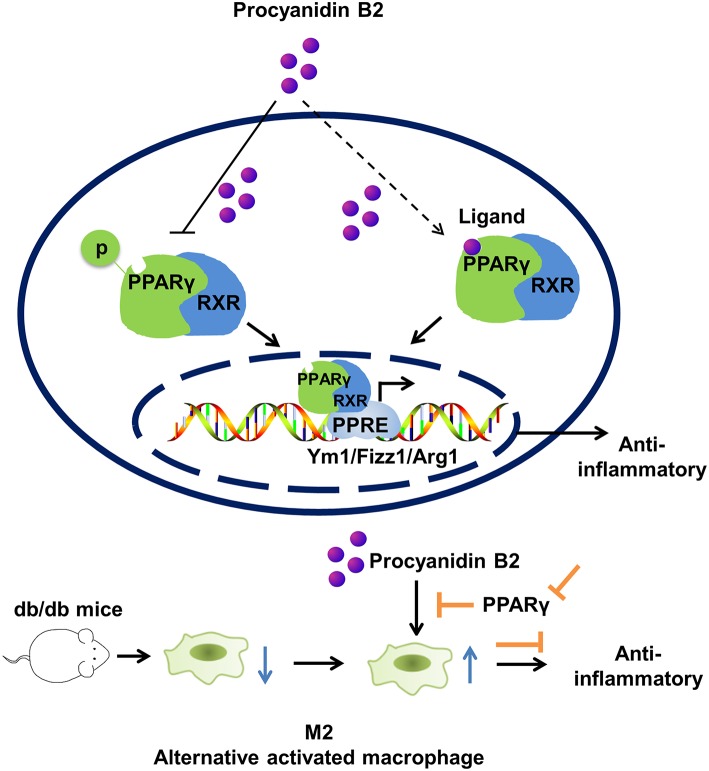
Effect of PCB2 on macrophage polarization. PCB2 enhanced the expressions of M2 markers (Arg1, Ym1, and Fizz1) and activated PPARγ activity in macrophages. Compared with db/m^+^ control mice, macrophages isolated from db/db diabetic mice showed an impaired M2 phenotype. PCB2 effectively restored the M2 polarization in a PPARγ-dependent manner.

Our findings demonstrated that PCB2 regulated macrophage M2 polarization in mouse macrophages *via* the activation of PPARγ (Figure 7). More importantly, our results demonstrated that PCB2 ameliorated obesity-related inflammation *via* a PPARγ-dependent up-regulation of Ym1, Arg1, and Fizz1. This finding revealed a novel mechanism underlying the beneficial effects of dietary procyanidins on metabolic and inflammatory diseases.

## Data Availability

The datasets generated for this study are available on request to the corresponding author.

## Ethics Statement

Animal care and experimental protocols were in accordance with the National Institutes of Health (NIH) Guide for the Care and Use of Laboratory Animals with the approval by the Animal Research Committee of Xi'an Jiaotong University.

## Author Contributions

LX and NW conceived the study, analyzed the data, and wrote the manuscript. YT performed most of the experiments and wrote the manuscript. CY, WM, XX, QY, LQ, JL, XN, and BL performed experiments and provided technical support. All authors had read and approved the manuscript.

### Conflict of Interest Statement

The authors declare that the research was conducted in the absence of any commercial or financial relationships that could be construed as a potential conflict of interest.
