# Supplementary material for: Procyanidin B2 Activates PPARγ to Induce M2 Polarization in Mouse Macrophages
Source: Front Immunol. 2019 Aug 7;10:1895. doi: 10.3389/fimmu.2019.01895 (PMC6693435; doi:10.3389/fimmu.2019.01895)
Supplement: Supplementary file 1 [file Data_Sheet_1.doc]

Supplementary Material

# Supplementary Figure and Tables

## Supplementary Figure


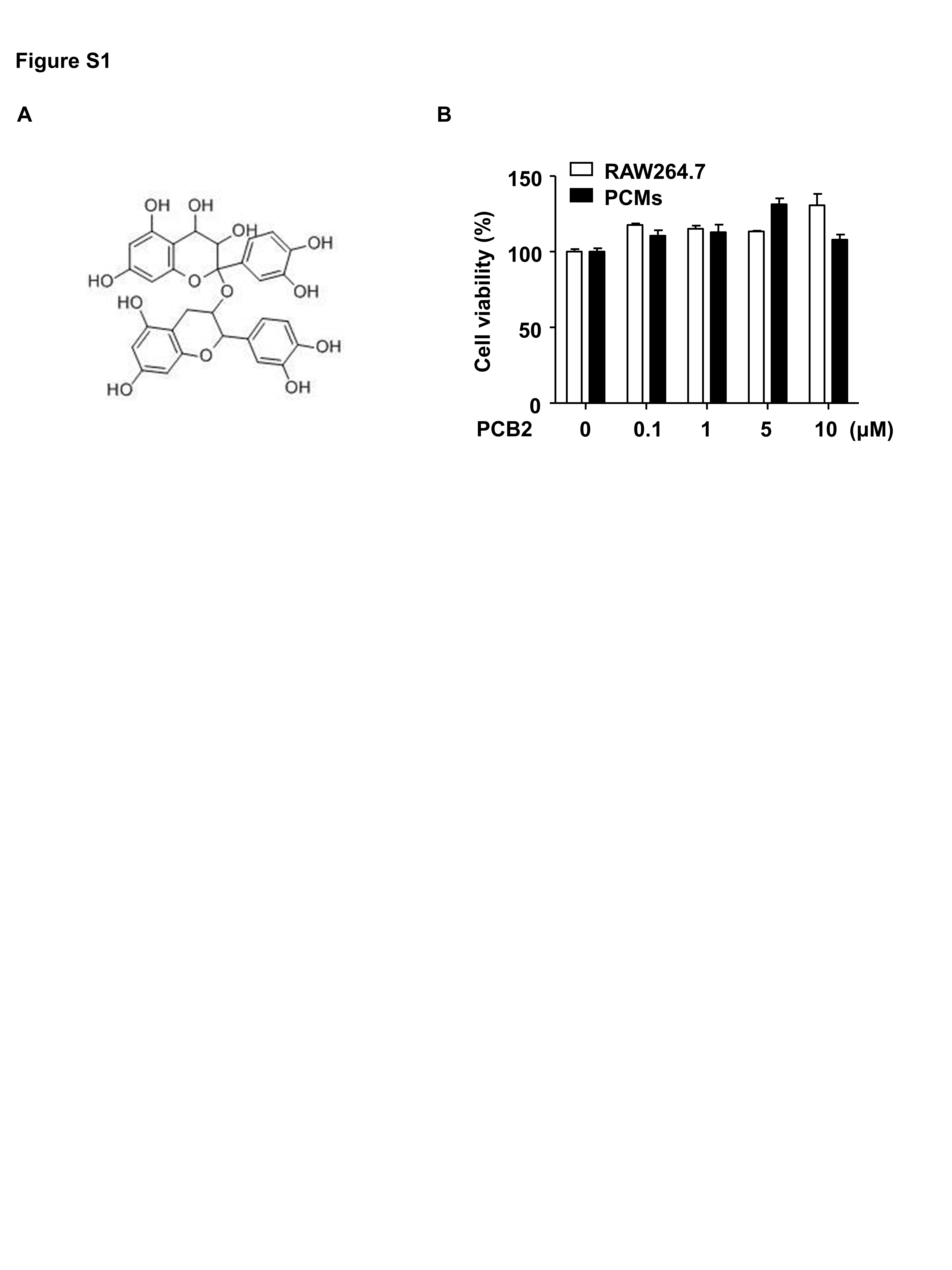


**Figure S1. There was no cytotoxic effect of PCB2 on RAW264.7 cells and PCMs. (A)** The chemical structure of PCB2. **(B)** RAW264.7 cells and PCMs were treated with indicated concentrations of PCB2 for 24 h, and cell viability was analyzed by MTT assay.


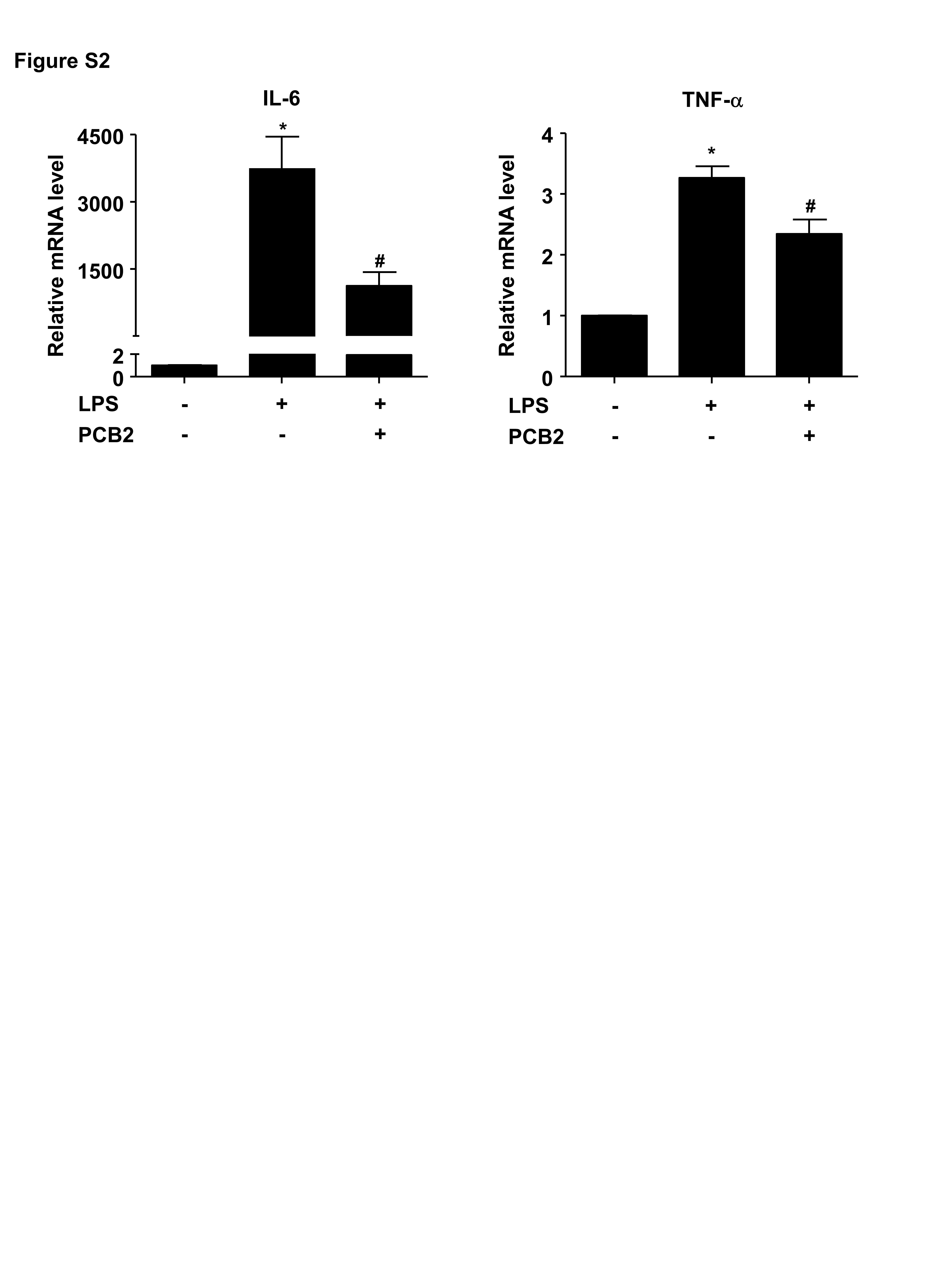


**Figure S2. PCB2 inhibited LPS-stimulated IL-6 and TNF-α up-regulation.** RAW264.7 cells were pretreated with PCB2 (10 μM) for 16 h, then exposed to LPS (100 μg/ml) for 8 h. The mRNA levels of IL-6 and TNF-α were assessed by qRT-PCR. Data were shown as mean ± SEM, n=3, **P*<0.05 *vs.* control and ^#^*P*<0.05 *vs.* PCB2.

**Supplementary Tables**

**Supplementary Table 1 Sequences of primers for qPCR**

| **Gene** | | **Sequences** |
| --- | --- | --- |
| **mYm1**  **mArg1**  **mFizz1**  **mTNF-α**  **mIL-6**  **mGAPDH** | Forward: AGAAGGGAGTTTCAAACCTGGT  Reverse: GTCTTGCTCATGTGTGTAAGTGA  Forward: ATGCTCACACTGACATCAACACTC  Reverse: CTCTTCCATCACCTTGCCAATCC  Forward: TCCAGCTAACTATCCCTCCACTGT  Reverse: GGCCCATCTGTTCATAGTCTTGA  Forward: GGCTGCCCCGACTACGT  Reverse: ACTTTCTCCTGGTATGAGATAGCAAAT  Forward: CTGCAAGAGACTTCCATCCAGTT  Reverse: AGGGAAGGCCGTGGTTGT  Forward: ACCACAGTCCATGCCATCAC  Reverse: TCCACCACCCTGTTGCTGTA | |

**Supplementary Table 2** Primers for ChIP assay

| **Gene** | | **Sequences** |
| --- | --- | --- |
| **mPPRE-Ym1-1**    **mPPRE-Ym1-2**  **mPPRE-Ym1-3**  **mPPRE-Ym1-4**  **mPPRE-Ym1-5**  **mPPRE-Ym1-6** | Forward: CCATTCTACCTTACTTCTCC  Reverse: TGGTCCTGTGAAGGTTCAAT  Forward: ACCTACTCCCCTTACCCTGT  Reverse: TACCTCCAGAGCTCCCAAGA  Forward: ACTTGTCAGTGAAGGCATACC  Reverse: GGAAAGAACCCAGATGTCCC  Forward: CCCATCGACTCACTCTACTT  Reverse: GCTGCTGGTATGTGATTCC  Forward: GGCTCATCACAGAAGGGGTT  Reverse: GGCCTACTTTCCTCTCAATGG  Forward: TGAAGTTCCACCCCTAGCTG  Reverse: TCACTTCCTCCCCTATCTCCT | |
| **mPPRE-Fizz1-1**  **mPPRE-Fizz1-2**  **mPPRE-Fizz1-3**  **mPPRE-Fizz1-4** | Forward: ATGACATTGCCTTCCCATGC  Reverse: TGTGTTGAGCTAGAGTCCCT  Forward: GGGCATTAAACATCACTTCTGC  Reverse: TGCTTCTTACAGTTGGCAGG  Forward: CACATCTCCTCTGACTAGC  Reverse: TGGAATCTGCTGCATGAG  Forward: AGCTCTGTGGGACTCTCTCT  Reverse: GCTGGAGGGAAAGAGCAAAG | |
| **mPPRE-Arg1-1**  **mPPRE-Arg1-2**  **mPPRE-Arg1-3,4**    **mPPRE-Arg1-5**  **mPPRE-Arg1-6** | Forward: GCAGGTTCTGTGTTGACCT  Reverse: ACCACCACCTGTCAATACC  Forward: GTAACTGGGAGAATACTTGG  Reverse: AGTTTGACCCGAAGAAC  Forward: TGGGTTCTTCGGGTCAAACT  Reverse: AATCGAAACGGAGCAATGGG  Forward: CACATCTCCTCTGACTAGC  Reverse: CTCATGTGAAGTGCTAGGC  Forward: TGGGGAGGTTCTGTTGACTC  Reverse: CATGCTCTCTCTGGGTTCCA | |
